# A fish intestinal epithelial barrier model established from the rainbow trout (*Oncorhynchus mykiss*) cell line, RTgutGC

**DOI:** 10.1007/s10565-017-9385-x

**Published:** 2017-03-01

**Authors:** Matteo Minghetti, Carolin Drieschner, Nadine Bramaz, Hannah Schug, Kristin Schirmer

**Affiliations:** 1 Eawag, Swiss Federal Institute of Aquatic Science and Technology, Überlandstrasse 133, 8600 Dübendorf, Switzerland; 2 Department of Integrative Biology, Oklahoma State University, Stillwater, OK USA; 3 School of Architecture, Microsystems Laboratory 4, EPF Lausanne, Lausanne, Switzerland; 4 School of Architecture, Civil and Environmental Engineering, EPF Lausanne, Lausanne, Switzerland; 5 Institute of Biogeochemistry and Pollutant Dynamics, ETH Zürich, Zürich, Switzerland

**Keywords:** Fish intestine, Polarized epithelium, Permeation, Ion regulation, Silver toxicity, In vitro model

## Abstract

The intestine of fish is a multifunctional organ: lined by only a single layer of specialized epithelial cells, it has various physiological roles including nutrient absorption and ion regulation. It moreover comprises an important barrier for environmental toxicants, including metals. Thus far, knowledge of the fish intestine is limited largely to in vivo or ex vivo investigations. Recently, however, the first fish intestinal cell line, RTgutGC, was established, originating from a rainbow trout (*Oncorhynchus mykiss*). In order to exploit the opportunities arising from RTgutGC cells for exploring fish intestinal physiology and toxicology, we present here the establishment of cells on commercially available permeable membrane supports and evaluate its suitability as a model of polarized intestinal epithelia. Within 3 weeks of culture, RTgutGC cells show epithelial features by forming tight junctions and desmosomes between adjacent cells. Cells develop a transepithelial electrical resistance comparable to in vivo measured values, reflecting the leaky nature of the fish intestine. Immunocytochemistry reveals evidence of polarization, such as basolateral localization of Na^+^/K^+^-ATPase (NKA) and apical localization of the tight junction protein ZO-1. NKA mRNA abundance was induced as physiological response toward a saltwater buffer, mimicking the migration of rainbow trout from fresh to seawater. Permeation of fluorescent molecules proved the barrier function of the cells, with permeation coefficients being comparable to those reported in fish. Finally, we demonstrate that cells on permeable supports are more resistant to the toxicity elicited by silver ions than cells grown the conventional way, likely due to improved cellular silver excretion.

## Introduction

The fish intestinal epithelium is an important environment-organism interface whose physiological functions include nutrient transport, osmoregulation and protection from environmental stressors, such as toxicants (Grosell et al. 2011). Research on the molecular and biochemical processes in the specialized intestinal cells fosters fundamental knowledge and provides impetus for improved fish health, as in aquaculture, and protection, as in environmental risk assessment. Yet, progress in this research has thus far largely relied on in vivo experiments. Sundh et al. (2014) and Grosell et al. (2007), for example, have used molecular, biochemical and immunohistochemical approaches on salmonids to understand the role of the fish intestine during the process of acclimation to seawater. However, these types of studies, while relying on a fully developed, functional epithelium, do not allow for controlled variation of experimental parameters on the level of the organ itself. Here, isolated perfused intestinal segments or sac preparations provide useful ex vivo experimental models. Indeed, gut sac preparations have been used to study intestinal absorption of metals in different fish species (Kwong and Niyogi 2009; Hogstrand et al. 2002; Handy et al. 2000). Again, the intestinal epithelium stays intact but the preparations are limited in their durability and only few experimental conditions can be tested per fish. Finally, freshly isolated intestinal epithelial cells can be used for short term in vitro investigations. Such an approach was, for example, chosen by Burke and Handy (2005) and Kwong and Niyogi (2012) to evaluate time- and concentration-dependent copper and cadmium accumulation. Yet, as cells are suspended, they lose their structure as organized epithelial cell layer, making the study of transport processes in the polarized cell layer impossible. Thus, to provide a much more accessible, tuneable, animal-free model for mechanistic studies on polarized fish intestinal cells, we here present details of the establishment of a novel fish epithelial barrier model based on the rainbow trout gut cell line, RTgutGC (Kawano et al. 2011).

In vivo, the epithelium of the intestine is organized in a monolayer composed of four basic cell types: absorptive epithelial cells, mucus-producing goblet cells, endocrine and immune cells (Jutfelt 2011). In addition, due to its intense self-renewal kinetics, the intestinal epithelium possesses stem cells residing in the intestinal crypt (Clevers 2013). The vast majority are epithelial cells also called enterocytes. These cells are polarized, differentiating an apical side facing the intestinal lumen, and a basolateral side facing the organism interior. One characteristic of the apical side are tight junctions, which seal the membranes between neighbouring cells to control diffusion through the intercellular space. Other features of fish enterocytes include the apical formation of microscopic membrane protrusions, so-called microvilli, as a means to increase the surface for absorption of nutrients, and the basolateral expression of the Na^+^/K^+^-ATPase (NKA), important for ion regulation and other physiological processes (Grosell et al. 2007; Marshall and Grosell 2006). Our focus in establishing the piscine in vitro intestinal barrier model was on characterizing the development of a polarized epithelium when growing the RTgutGC cell line as monolayer on a permeable support. Similar methodology has previously led to the development of models of the human intestinal epithelium based on Caco-2 cells (Sambuy et al. 2005) and of the gill epithelium based on primary gill cells (Bury et al. 2014).

Referring to the first characterization of the RTgutGC cell line upon routine maintenance on solid cell culture support (Kawano et al. 2011), this cell line was initiated from a primary culture derived from the distal portion of the gut of a small, healthy female rainbow trout. The cells appear to have immortalized spontaneously, are heteroploid and possess an epithelial-like morphology. Moreover, they were reported to express alkaline phosphatase activity, a commonly considered marker of enterocyte differentiation, when cultures were initiated at very high cell densities (Kawano et al. 2011). We therefore started to explore to what extent the RTgutGC cells develop a functional epithelium if cultured in a two-compartment system on permeable cell culture support, where an apical and a basolateral side can be mimicked to promote polarization and enable transport-dependent studies at this interface. We have previously shown that these cells can be cultured on commercial inserts with pore sizes ranging from 0.4 to 3 μm and that membranes with the 3-μm pore size are amenable to study the transport of nanoparticles across the epithelial cell layer (Geppert et al. 2016).

We here report on the step-by-step establishment of the intestinal epithelial cell monolayer on 0.4-μm pore size membranes, the selected structural and functional features characterizing this cell layer and its response to a physiological (acclimation to increased salinity) and a toxicological (silver ions) stimulus. Acclimation to increased salinity was selected because of its physiological importance in anadromous fish, such as rainbow trout, and because the intestine has a key role in this process (Kültz 2015). Exposure to silver ions was chosen because silver is a toxic metal known to be able to cross the intestinal barrier, likely due to copper transporters (Behra et al. 2013).

## Material and methods

### RTgutGC cell culture

#### Routine cell culture

RTgutGC cells were routinely cultured essentially as described by Kawano et al. (2011). Briefly, cells were grown in 75-cm^2^ flasks (TPP, Trasadigen, Switzerland) in Leibovitz’s L-15 Medium without phenol red (no. 21083-027, Invitrogen, Basel, Switzerland) supplemented with 5% foetal bovine serum (FBS, Gold, PAA Laboratories GmbH, Austria) and 1% gentamicin (PAA Laboratories GmbH, Austria, 10 mg/mL). Cells were maintained at 19 °C in normal atmosphere and split into two flasks once confluent, which was every 2 to 3 weeks. Confluent cells were washed twice with Versene (Invitrogen, Basel, Switzerland), and cells were detached using trypsin (0.25% in phosphate-buffered saline, PBS, Biowest, Germany).

#### Cell culture on permeable membranes

RTgutGC cells were seeded onto commercially available, transparent tissue culture inserts for multiwell plates (pore size = 0.4 μm; polyethylene terephthalate [PET] from Greiner Bio-One, Germany). Insert size was chosen depending on the application (see the following): 0.33-cm^2^ inserts (for 24-well plates) for cell viability, confocal microscopy and electron microscopy; 1.13-cm^2^ inserts (for 12-well plates) for permeability and quantitative RT-PCR (qPCR) analyses; and 4.52 -cm^2^ inserts (for 6-well plates) to determine the concentration of silver in cells after a 24 h exposure. Initial experiments with or without fibronectin showed that the fibronectin promoted faster attachment and a more homogeneous distribution of cells—fibronectin coating was therefore used throughout. To accomplish the coating, inserts were incubated at room temperature for 2 h with fibronectin (50 μg/mL; Roche Applied Science, Basel, Switzerland) in PBS (Bioswisstec AG, Schaffhausen, Switzerland) then washed once with PBS prior to adding cells. To initiate cultures on the inserts, cell suspensions in L-15/FBS were added in 300, 1000 and 3000 μL for 24-, 12- and 6-well inserts (referred to as apical compartment), respectively. These were then placed in the corresponding culture wells (referred to as basolateral compartment), which were subsequently filled with, respectively, 1000, 1700 and 3400 μL of L-15/FBS. When both the apical and the basolateral compartment was filled with the same medium, such as L-15/FBS as described previously, the set-up is termed “symmetrical”. In case that the medium composition differed in the two compartments, the set-up is termed “asymmetrical” (see below).

All experiments were carried out with a starting cell density of 62,500 cells/cm^2^, counting cells with a CASY® cell counter (Casy model TTC, Schärfe System GmbH, Reutlingen, Germany). After testing different densities initially, this cell density was found to lead to the formation of a confluent monolayer within 1 day, reducing culture time, but at the same time be low enough to avoid the formation of multiple cell layers. Culture medium was fully replaced by fresh medium in both the apical and basolateral compartment approximately every 7 days. The formation of the barrier was assessed based on transepithelial electrical resistance (TEER), the evolution and localization of zona occludens (ZO-1), NKA and f-actin proteins by immunocytochemistry/confocal microscopy and non-invasive cell viability measurements (see the following section). Moreover, electron microscopy was used to further confirm the presence of tight junctions and desmosomes and to verify that the cell barrier consisted of only a single layer of cells.

### Measurement of TEER

TEER measurements were done prior to medium change using an epithelial tissue voltohmeter (EVOMX; World Precision Instruments) fitted with chopstick electrodes (STX-2) and surface-normalized values calculated according to the manufacturer’s instructions. Measurements across inserts with medium but no cells served as control, and resulting values were subtracted from results with cells.

### Immunocytochemistry and confocal microscopy

To initiate imaging, inserts were washed twice with PBS. Cells were fixed by incubation in a solution of 3.7% paraformaldehyde (Sigma-Aldrich, Switzerland) in PBS for 15 min at room temperature. Permeabilization was achieved by incubating the cells in a solution of 0.2% (*v*/*v*) Triton (Sigma-Aldrich, Switzerland) in PBS for 30 min at 4 °C. The “image-it” solution (Molecular Probes, Invitrogen, Switzerland) was used for blocking. For detection of zonula occludens, a monoclonal antibody (ZO-1) conjugated to Alexa Fluor® 488 was obtained from Invitrogen (Molecular Probes, Invitrogen, Switzerland) and applied at 5 μg/mL in PBS. Monoclonal sodium-potassium ATPase α5-subunit antibody (NKA, Developmental Studies Hybridoma Bank, The University of Iowa, USA) was applied at 5 μg/mL in PBS. Alexa Fluor® 488 conjugated anti-mouse secondary antibodies (Molecular Probes, Invitrogen, Switzerland), used in combination with the NKA primary antibody, were also applied at 5 μg/mL in PBS. Primary antibody incubation was performed overnight at 4 °C, while incubation with secondary antibody and the f-actin staining (Rhodamine phalloidin, Molecular Probes, Invitrogen, Switzerland), applied at 2 unit/mL in PBS, were done for 1 h at room temperature. Nuclear counter staining was achieved by incubating cells with 300 nM DAPI (Molecular Probes, Invitrogen, Switzerland) for 5 min at room temperature just before imaging. Finally, the PET membrane holding the cells was washed several times with PBS, excised from the insert, mounted on a microscope slide (Thermo Scientific, Switzerland) and covered with ProLong Antifade solution (Molecular Probes, Invitrogen, Switzerland).

Imaging was performed using a Leica SP5 (Wetzlar, Germany) upright confocal laser scanning microscope, equipped with the acquisition software LAS AF 2.6.v. The ×63 OIL (NA 1.4) objective was used. Images at each wavelength (405 nm for DAPI, 488 nm for Alexa Fluor® 488 conjugated secondary antibodies and 543 nm for Rhodamine phalloidin) were acquired sequentially. For visualization, Z-stacks were recorded; to avoid bleaching of the fluorescent dyes, step size was manually set at 0.6 μm. Images 1.2 μm from the top (apical) or bottom (basolateral) side were recorded. Signals from five fields of view (FOVs; 5337 ± 350 μm^2^) were analysed by the co-localization module of the LAS AF 2.6v software. Co-localization of ZO-1 and f-actin varied along the Z dimensions; however, only the highest value of co-localization was recorded for each FOV. Side views were generated using 3D rendering module from the Imaris software (Bitplane, v. 7.6.5, Switzerland).

### Electron microscopy

#### Chemical fixation

The PET membrane with the cells was carefully excised from the insert. The samples were fixed in phosphate-buffered 2.5% glutaraldehyde, post-fixed in 1% osmium tetroxide and block stained with 1% uranyl acetate then dehydrated in a graded ethanol series and finally embedded in Epon resin (EMbed 812, EMS). After polymerization at 60 °C, ultrathin sections were cut on an ultramicrotome (Reichert Ultracut S) perpendicular to the cell monolayer. Due to different material properties of resin and membrane, some folding occurred. For scanning electron microscopy (SEM), 100-nm sections were transferred onto silicon chips: no post-staining was applied. Micrographs were taken on a FEI Magellan 400 at 2 kV the backscatter electron signal. Large area scans were performed by stitching individual image tiles.

Chemical fixation is a simple and fast preparation method, but the integrity of membranes is not guaranteed, and therefore, the structure might be prone to artefacts. Therefore, findings were compared with specimen prepared by freeze substitution followed by TEM analyses.

#### Freeze substitution

The PET membrane with the cells was carefully excised from the insert. With a biopsy puncher, 5-mm discs were punched out and transferred into a sandwich made of two aluminium specimen carriers with a sample cavity of 100 μm of depth. The carriers were previously covered with a thin layer of hexadecane to facilitate the removal of the frozen sample during the follow-up preparation and filled with PBS buffer. The sandwiches were high-pressure frozen (Bal-Tec; HPM 100) and transferred into a freeze substitution unit (homemade with Tecon Temperature Controller) pre-cooled to −90 °C for the substitution in acetone mixed with 1.6% OsO4, 0.2% UrAc and 5% water. The substitution was done at −90 °C for 3 h, −70 °C for 2 h and −35 °C for 12 h, washed in acetone and embedded in HM20 before polymerizing under UV. Ultrathin sections (50 nm, Reichert Ultracut S) were stained with uranyl acetate and lead citrate and analyzed using TEM (FEI; Morgagni) at an acceleration voltage of 100 kV.

### Assessment of cell viability

Non-invasive assessment of cell viability was accomplished by means of the fluorescent cell viability indicator dye, Alamar Blue, essentially as described by Schirmer et al. (1997). Alamar Blue is a commercial preparation of the dye resaruzin, which enters the cells and is reduced to resorufin by mitochondrial, microsomal and cytosolic oxidoreductases (O’Brien et al. 2000). Cell viability was assessed as one of the parameters of the intactness of RTgutGC cells cultured in inserts. In the case of exposure of cells to AgNO_3_ (see the following section), cell viability was additionally assessed in cells grown conventionally on the bottom of 24-well plates (Greiner Bio-One, Germany).

#### Cells cultured in inserts (0.33 cm^2^)

At the time of assessment, the exposure medium was aspirated first from the basolateral and then the apical compartment and cells were washed once with PBS. Then, 100 μL of a 5% Alamar Blue solution was added to the apical compartment; the basolateral compartment was left empty. Cells were incubated in the dark at 19 °C for 30 min, and then fluorescence was recorded from the top with a Tecan Infinite 2000 multiwell plate reader (Switzerland) at an excitation/emission wavelength of 530 and 595 nm, respectively. To account for the small area of the insert and the fact that the position of inserts may shift slightly in the wells, nine positions were measured. These positions reflect the fluorescence in the centre of the inserts with very small variations among them. The resulting average fluorescent units (F.U.) were corrected for background fluorescence measured in inserts without cells. In one experiment, the Alamar Blue fluorescence values were converted to cell number based on a separately prepared standard curve of F.U. vs. insert cell number according to Ganassin et al. (2000).

#### Cells cultured in wells of 24-well plates

To compare the response of cells grown in inserts to those grown conventionally on the bottom of culture wells upon exposure to a toxicant, AgNO_3_, cells were plated in the 24-well plates and cell viability was assessed as described previously with two modifications. First, the volume of Alamar Blue added was 400 μL; and second, fluorescence was measured based on a single read in the middle of the well as described by Schirmer et al. (1997).

### Permeation of fluorescently labelled molecules

To assess the tightness and integrity of the RTgutGC cell layer, three fluorescently labelled dyes of different molecular size were used: Lucifer Yellow (LY, potassium salt, MW = 522 Da; Thermo Fisher Scientific, Switzerland), Dextran FD4 (FD4, MW = 4000 Da; Sigma-Aldrich; Buchs, Switzerland) and Dextran FD40 (FD40, MW = 40,000 Da; Sigma Aldrich; Buchs, Switzerland). Since excitation and emission maxima of the dyes overlap (*λ*_ex/em_ LY = 450/520 nm, *λ*_ex/em_ FD4/FD40 = 485/544 nm), they were applied and analyzed in separate preparations of cells. Dyes were dissolved in the medium used during permeation studies (see below) at a final concentration of 50 μg/mL for LY and 1 mg/mL for FD4/FD40.

To initiate the permeation assay, TEER values of cultures grown for 21–28 days in 1.13-cm^2^ (12-well plate) inserts were measured first. Then, cells were pre-incubated with the specified medium (apical [1 mL]: L-15/FBS, L-15/ex, seawater (SW) or freshwater (FW); basolateral [1.7 mL]: L-15/FBS or L-15/ex) for 30 min at 19 °C and TEER measurement was repeated. Thereafter, dyes were applied to the apical (=donor) compartment and plates were incubated at 19 °C for up to 72 h. At different time points, the solution in the basolateral (=receiver) compartment was carefully mixed and then 50 μL was withdrawn from two different positions. The 50 μL aliquots were transferred to a 96-well plate, and the fluorescence was detected immediately at the respective emission and excitation wavelength. In the beginning, in the middle and at the end of exposure, a sample from the apical compartment was taken to verify the stability of the dye concentration in the apical compartment. The fluorescence data were transformed to amount permeated dye based on the linear regression of separately prepared standard curves.

The removed sample volume was taken into account by adjusting the total receiver volume after each measurement. The apparent permeability *P*_app_ [cm/s] was calculated according to Hubatsch et al. (2007, protocol 2) as follows:

1 $$ {P}_{\mathrm{app}}=\left(\frac{dQ}{dt}\right)\times \left(\frac{1}{A\times {C}_0}\right) $$

with $\left(\frac{dQ}{dt}\right)$ being the steady state flux [μmol s^−1^], *A* the insert surface area [cm^2^] and *C*_0_ the initial concentration [μM] in the apical compartment. For this way of analysis, the receiver concentration should not exceed 10% of the donor concentration in order to maintain steady-state conditions throughout the experiment (Hubatsch et al. 2007).

### Expression of sodium-potassium ATPase

NKA was assessed in two ways: (1) based on enzyme activity in conventionally grown RTgutGC cells compared to activity found in intestinal and gill tissue and (2) based on messenger RNA (mRNA) abundance using qPCR in cells grown in inserts.

#### Enzyme activity measurements

The basic protocol for NKA activity was performed according to McCormick (1993) with some modifications for the RTgutGC cell line. Tissues from rainbow trout were obtained from Machrihanish fish farm, Campbeltown, UK. Gills (four–five gill filaments) from four individuals, and anterior intestine (between the pyloric ceca and the ileocecal valve) and posterior intestine (between the ileocecal valve and the anus) from three individuals, were dissected and immediately frozen in liquid nitrogen. Modifications were required to adapt the method to the RTgutGC cell culture. The ratio of cell number to the SEI buffer (150 mM sucrose, 10 mM EDTA, 50 mM imidazole, pH 7.3) was optimized: 8.3 × 10^6^ cells (usually obtained from one 75-cm^2^ flask) were re-suspended in 200 μL of SEI, which corresponds to an optimal amount of protein for the assay (~30–50 μg protein/10 μL). Thereafter, samples were immediately frozen at −80 °C.

To initiate NKA activity analyses, tissues or cells were thawed on ice. With regard to the intestinal tissue, gut enterocytes were collected via scraping of the mucosa layer from the serosa muscolar layer with a glass microscope slide. Tissues (about 10 mg per analysis) were then homogenized in 100 μL ice-cold SEI buffer using a mechanical homogenizer ULTRA-TURRAX® whereas RTgutGC cells (8.3 × 10^6^ in 200 μL SEI) were homogenized via sonication (three pulses for 5 s; LabSonic®).

#### RNA extraction, cDNA synthesis and quantitative PCR

Total RNA was extracted by adding 600 μL of TRIzol® Reagent (Invitrogen/Gibco, Germany) directly onto the RTgutGC cell monolayer grown on 1.13-cm^2^ (12-well) inserts. The phase separation was performed using Phase Lock heavy tubes (5prime, USA), adding an additional ethanol precipitation step (1:10 volumes of sodium acetate 3 M and 1:3 volumes of ethanol) supplemented with 1 μg/μL RNase free glycogen (Fermentas, Switzerland). The purified total RNA was then DNase treated using the TURBO DNase Kit (Invitrogen/Ambion, Germany) following the manufacturer’s instructions. Quantity and quality of isolated RNA was determined by spectrophotometry with an ND-1000 NanoDrop (Labtech Int., East Sussex, UK) and by electrophoresis using 0.5 μg of total RNA in a 1% agarose gel. The ratio 260:280 was always more than 1.9, and an average of ~3 μg of total RNA was purified from each insert. Complementary DNA (cDNA) synthesis was performed using the Advantage RT-for-PCR Kit (Clontech, Switzerland) from 1 μg of total RNA and applying 500 nM of random hexamer and oligo-dT following the manufacturer’s instruction. Subsequent to the cDNA synthesis, the samples were diluted ten times in nuclease-free water (Sigma-Aldrich, Switzerland) for qPCR analysis. Primers for qPCR were designed using Primer-BLAST (http://www.ncbi.nlm.nih.gov/tools/primer-blast/) as detailed in Table 1. Oligonucleotide primers for the target gene ATP1a1 (coding for NKA) and the reference gene, elongation factor 1 alpha (EF1α), were used at 0.3 μM with 1/40 of the cDNA synthesis reaction (5 μL of a 1:10 dilution) and 10 μL of SYBR Green Premix Taq II (Takara, USA), in a total volume of 20 μL. Each sample was run in triplicate using an Applied Biosystems 7500 Fast Real-Time PCR System thermocycler. Quantitative PCR cycling conditions were as indicated in the Takara instruction manual: 1 cycle at 95 °C for 30 s followed by 40 cycles at 95 °C for 5 s and 60 °C for 1 min. After 40 cycles, a melt curve was generated by measuring sample fluorescence during heating from 60 to 95 °C. The specificity of reactions was checked by inspecting melting curve profiles and by sequencing of amplicons from a random selection of samples. Determination of amplification efficiencies was measured using a dilution series of a pool of cDNA samples. All amplification efficiencies were over 93%. Quantification was achieved by a parallel set of reactions containing standards consisting of serial dilution of spectrophotometrically determined, linearized plasmid containing the above-mentioned cDNA sequences. Cloning of PCR products was achieved using the TOPO® TA Cloning® Kits (Invitrogen, UK). Normalization of copy number across biological samples was achieved by dividing the copy number of the target gene ATP1a1 to the copy number of the reference gene EF1α. To confirm cDNA sequences, sequencing was performed using a Beckman 8800 autosequencer, and the Lasergene SeqMan software (DNASTAR) was used to edit and assemble DNA sequences.

**Table 1:** Primers used for qPCR

Gene name	Forward primer 5″→3″	Reverse primer 5″→3″	Repository ID^a^
ATP1a1	TGTGGCCGTCTTTCTGGGCATG	AGCAAATGGTGGAGGTGGAGCC	NM_001124459
EF1α	ATATCCGTCGTGGCAACGTGGC	TGAGCTCGCTGAACTTGCAGGC	NM_001124339

^a^GenBank (http://www.ncbi.nim.nih.gov/)

### Response of cells to buffers mimicking saltwater or freshwater

To test if cells would sustain exposure to medium that more closely reflects the milieu of the intestinal lumen, L-15/FBS culture medium in the apical compartment was replaced by L-15/ex. This is a simplified version of the complete L-15 culture medium: it contains only the salts, galactose and pyruvate of the original medium composition (Schirmer et al. 1997; Table 2).

**Table 2:** Composition of the different buffers to create asymmetrical culture conditions

Inorganic salts and other components (mM)	L-15/ex^a^	FW^b^	SW^b^
NaCl	155	151	69.0
KCl	6.08	3.00	5.00
MgSO_4_	1.88	0.88	77.5
MgCl_2_	2.38		22.5
CaCl2	1.44	1.00	5.00
NaHPO_4_	1.52	0.50	
KH2PO_4_	0.50	0.50	
NaHCO_3_		5.00	
Glucose		5.00	
Galactose	5.70		
Pyruvate	5.70		
HEPES (free acid)		3.00	
HEPES (Na salt)		3.00	
Ionic strength^b^ (mmol/kg)	173	158	289
Osmolality (mmol/kg)	354	339	283
pH	7.2	7.6	7.6

^a^According to Schirmer et al. (1997)

^b^Freshwater (FW), saltwater (SW): these buffer compositions were chosen to reflect the luminal composition after exposure of fish in freshwater or saltwater on the basis of Genz et al. (2011)

^c^Ionic strength was calculated using the software Visual MINTEQ. Osmolality was measured using an osmometer (Vapro 5600, Wescor, USA)

To specifically explore how cells respond to freshwater vs. seawater conditions, buffers were prepared to mimic luminal and extracellular fluids of fish in vivo acclimating to SW and FW according to Genz et al. (2011) (Table 2). While the FW buffer is very similar to L-15 medium in terms of salt composition, the SW medium has 2.2-fold less sodium and 41 and 3.4 more magnesium and calcium, respectively. The cell layer was established in complete media under symmetrical conditions on inserts for 3 weeks as described previously. The two buffers were then added to the apical compartment whereas the basolateral compartment was maintained with complete medium.

### Response of cells to silver ion exposure

#### Exposure

The response to AgNO_3_ exposure was evaluated in confluent conventional cell monolayers in 24-well plates (seeded at 62,500 cells/cm^2^ and incubated for 48 h prior to exposure) and in cells grown in 0.33 cm^2^ (24-well) inserts as described previously. To determine toxicity of AgNO_3_, cells were washed twice with PBS. Then, 1000 or 300 μL of AgNO_3_, dissolved at concentrations ranging from 0.08 to 50 μM in L-15/ex, were applied to cells in the wells or inserts, respectively. L-15/ex was also used in the bottom compartment of the insert (symmetrical conditions). Symmetrical conditions with protein-free media were chosen to avoid scavenging of silver ions by medium proteins (Minghetti and Schirmer 2016). Cells were incubated for 24 h at 19 °C in the dark. Following exposure, cell viability was assessed as described previously and expressed as percentage of non-exposed controls.

#### Cell internal metal accumulation

Intracellular silver concentrations were measured by ICP-MS (Element 2 high-resolution sector field ICP-MS, Thermo Finnigan, Germany). Thus, RTgutGC cells were seeded as described above in six-well plate inserts (4.52 cm^2^, 3 mL volume) or in wells of six-well plates to achieve a conventional monolayer. Cells were exposed to 400 nM AgNO_3_ in L-15/ex for 24 h. This concentration was chosen to be sufficiently high for ICP-MS analysis and sufficiently low to maintain cell viability within 100 ± 3% as assessed by Alamar Blue. To ensure removal of loosely bound silver, cells were then washed twice with a solution of 0.5 mM cysteine in PBS. Cells were lysed by applying 1 mL of 50 mM NaOH and incubated at room temperature for 2 h. An aliquot of the cell lysate was used for protein quantification using the modified Lowry assay (Thermo Scientific, USA) and bovine serum albumin as a standard. For metal determination, samples were desiccated using a concentrator (Concentrator plus, Eppendorf, Germany) and digested adding 2 mL of 65% HNO_3_ and 0.5 mL of 30% H_2_O_2_ in a high-performance microwave digestion unit (MLS 1200 MEGA, Switzerland) at a maximum temperature of 195 °C for 20 min. The digest was then diluted 50 times and measured. The recovery of AgNO_3_ was ≥99%. The reliability of the measurement was determined using specific water references (M105A, IFA System, Austria).

### Data analysis

Unless otherwise indicated, data were analysed and plotted using GraphPad Prism ver. 5.0, San Diego, CA. Statistical analysis methods applied are described in the corresponding figure legends.

## Results

### Establishment of a monolayer of intestinal cells

**Fig. 1:**
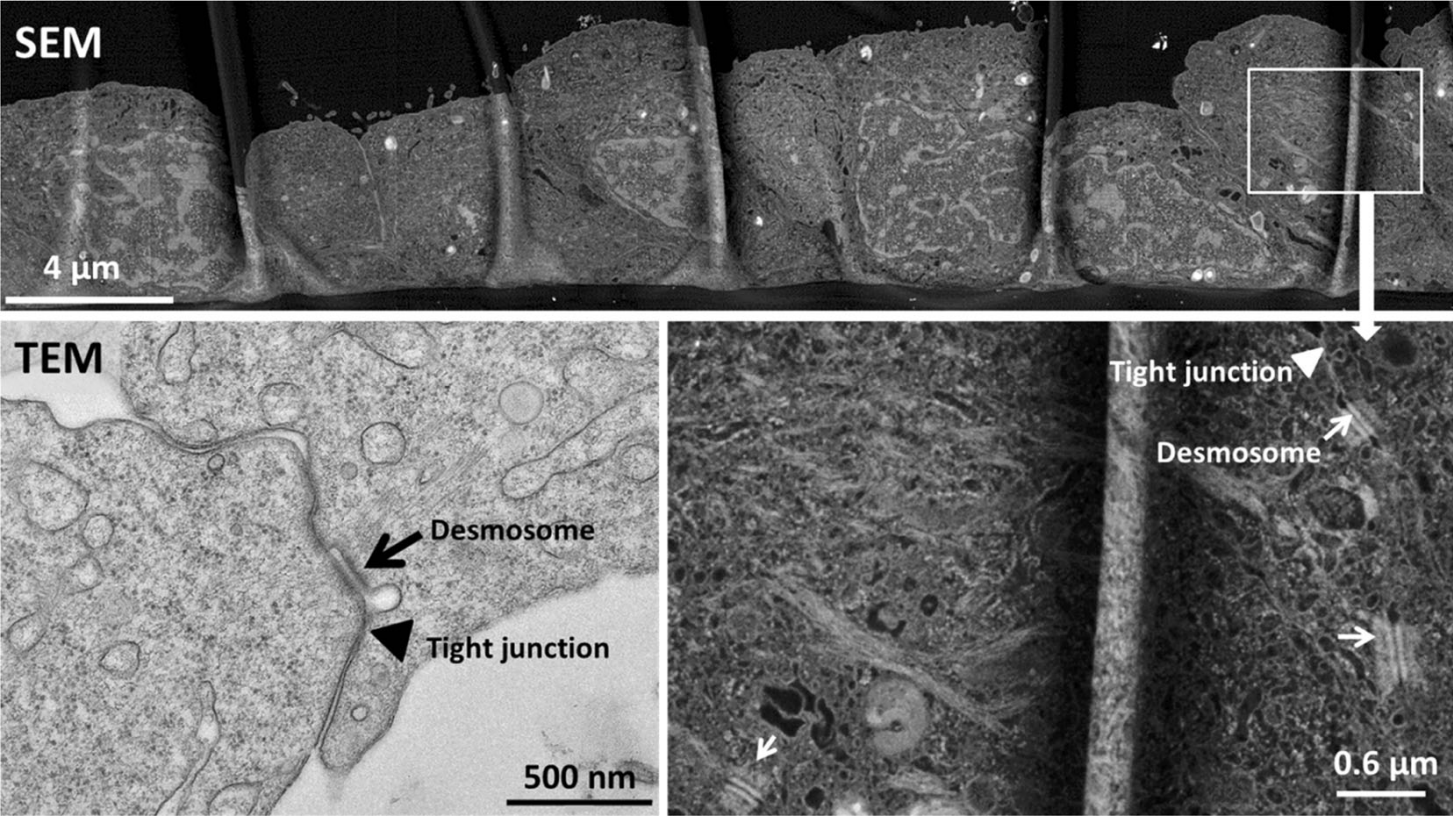
Image of RTgutGC cells grown for 21 days on permeable membranes (0.33 cm^2^—inserts of 24-well plates; starting cell density: 62,500 cells/cm^2^). The image obtained by scanning electron microscopy (SEM), demonstrating that cells are present as monolayer. A magnification of a portion of the monolayer is shown in *the lower right image*. The *lower left image* was obtained by transmission electron microscopy (TEM). The position of tight junctions, located at the apical side of the lateral membrane, is marked with a *triangle*; desmosomes are indicated with an *arrow*

**Fig. 2:**
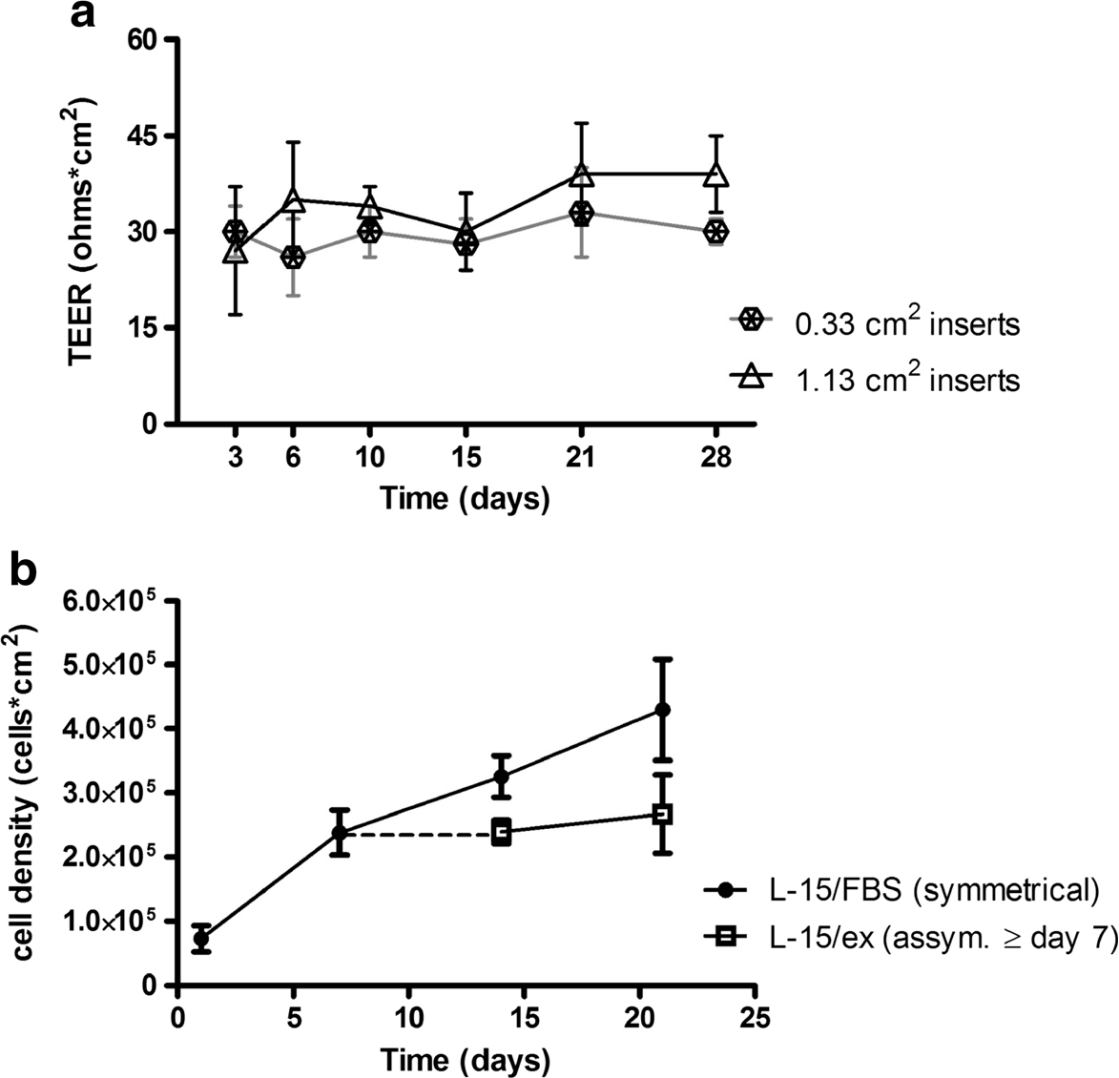
Development of transepithelial electrical resistance (TEER) and RTgutGC cell density when cultured on the permeable membranes over time. **a** Evolution of TEER in inserts of two different sizes: 0.33 (inserts of 24-well plates) and 1.13 cm^2^ (inserts of 12-well plates). Data shown are mean ± standard deviation from three to nine independent replicates. **b** Cell density over time in 0.33-cm^2^ (24-well plate) inserts. Cells were either cultured continuously under symmetrical conditions in L-15/FBS (*black circles*) or were switched to asymmetrical conditions at day 7, where the basolateral compartment continued to receive L-15/FBS but the apical compartment contained L-15/ex (*white squares*). Data represent the mean of three independent experiments ± standard deviation with three technical replicates (three inserts) in each

A cell density of 62,500 cells/cm^2^ and a culture period of about 21 days with symmetrical culture conditions were found to consistently yield a dense, single epithelial cell layer on the permeable membranes (Fig. 1—SEM image). We also cultured cells for 28 days or even longer with similar results, although a double cell layer was occasionally observed in some locations after ≥28 days. We started to measure TEER after the cells had been in culture for 3 days to first allow the cells to attach and adjust to their new culture environment. TEER values reached between 30 to 50 Ω × cm^2^ (Fig. 2a, Fig. 6c/d) from 3 to 28 days with little evolution of TEER values over time. Over the same period, cell number increased by about sixfold when monitored for up to 21 days (Fig. 2b, black circles). When we exchanged the cell culture medium in the apical chamber with the simple buffer, L-15/ex, after the first week of culture, cells stopped proliferating but remained viable (Fig. 2b, white squares). These results demonstrate that the culture system is stable under asymmetrical conditions with a simple buffer apically, which can be taken to more closely resemble the conditions in vivo under food deprivation (Minghetti and Schirmer 2016).

### Structural and functional features of the intestinal cell layer

**Fig. 3:**
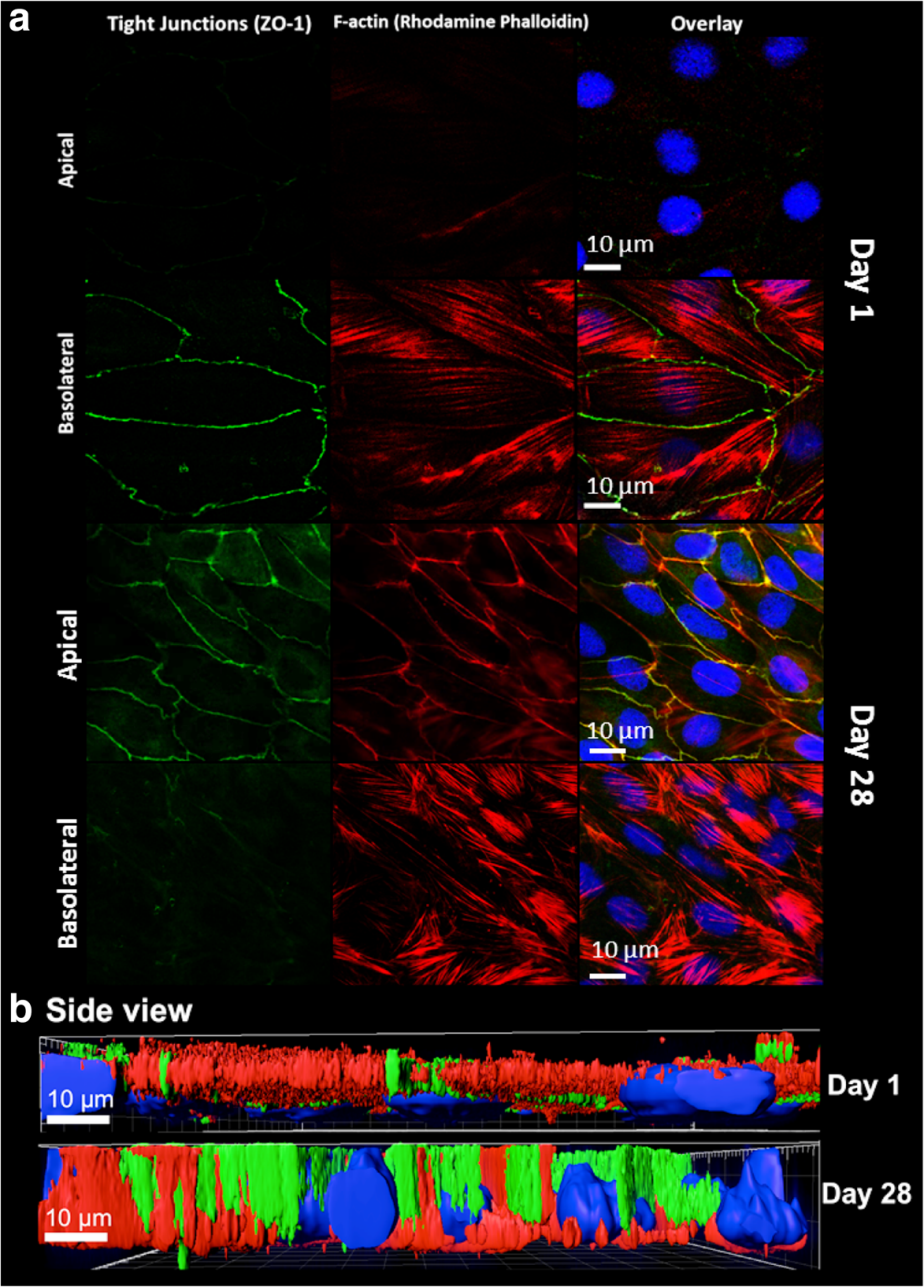
Immunocytochemical analysis of RTgutGC cells grown for 1 and 28 days on permeable membranes (0.33 cm^2^—inserts of 24-well plates; starting cell density: 62,500 cm^2^). Following fixation and permeabilization, cells were stained to highlight important cellular features: tight junctions (ZO-1 conjugated with Alexa Fluor 488; *green*), f-actin (Rhodamine phalloidin; *red*) and nuclei (DAPI; *blue*). Apical and basolateral images were obtained via Z-stack (**a**). Side view (**b**) was generated analysing Z-stacks using the Imaris software (Bitplane, v. 7.7) for 3D rendering. *Scale bar* is 10 μm. Cells were imaged using a Leica SP5 confocal microscope

Scanning and transmission electron microscopy analyses of RTgutGC cells grown on inserts show the formation of cellular structures involved in cell-cell adhesion typical of epithelia, namely desmosomes and tight junctions (Fig. 1—TEM image). Specific antibody staining further confirms the presence of the tight junction protein ZO-1 (Fig. 3). As illustrated in Fig. 3a, cells polarize over time: between day one and day 28, ZO-1 proteins apparently move to the apical side; likewise, f-actin develops an apical structure co-localizing with ZO-1 while basolaterally f-actin stress fibres are present from day one. This process was confirmed in co-localization analysis (Table 3). A side view image moreover reveals that the cells become taller as the cell layer becomes more compact: cells increase in height from about 6 μm on day one to about 11 μm within 21 days (Table 3; Fig. 3b).

**Table 3:** Co-localization of f-actin and ZO-1 and Z-dimension over time

Time (days)	Max % co-localization ZO-1/f-actin^a^	Z-dimension^a^ (μm)
1	10.9 ± 2.8	6.0 ± 1.2
3	15.2 ± 10.3	8.4 ± 1.4
6	14.8 ± 3.7	9.9 ± 0.5
10	18.8 ± 7.6	9.9 ± 1.9
15	31.7 ± 10.4	9.7 ± 0.4
21	56.4 ± 12.7	11.4 ± 1.7
28	55.9 ± 7.9	11.0 ± 0.7

^a^Values are means ± SD of five fields of view (FOV: 5337 ± 350 μm^2^) obtained using the Leica software LAS AF v. 2.6.3 (see also Fig. 3)

**Fig. 4:**
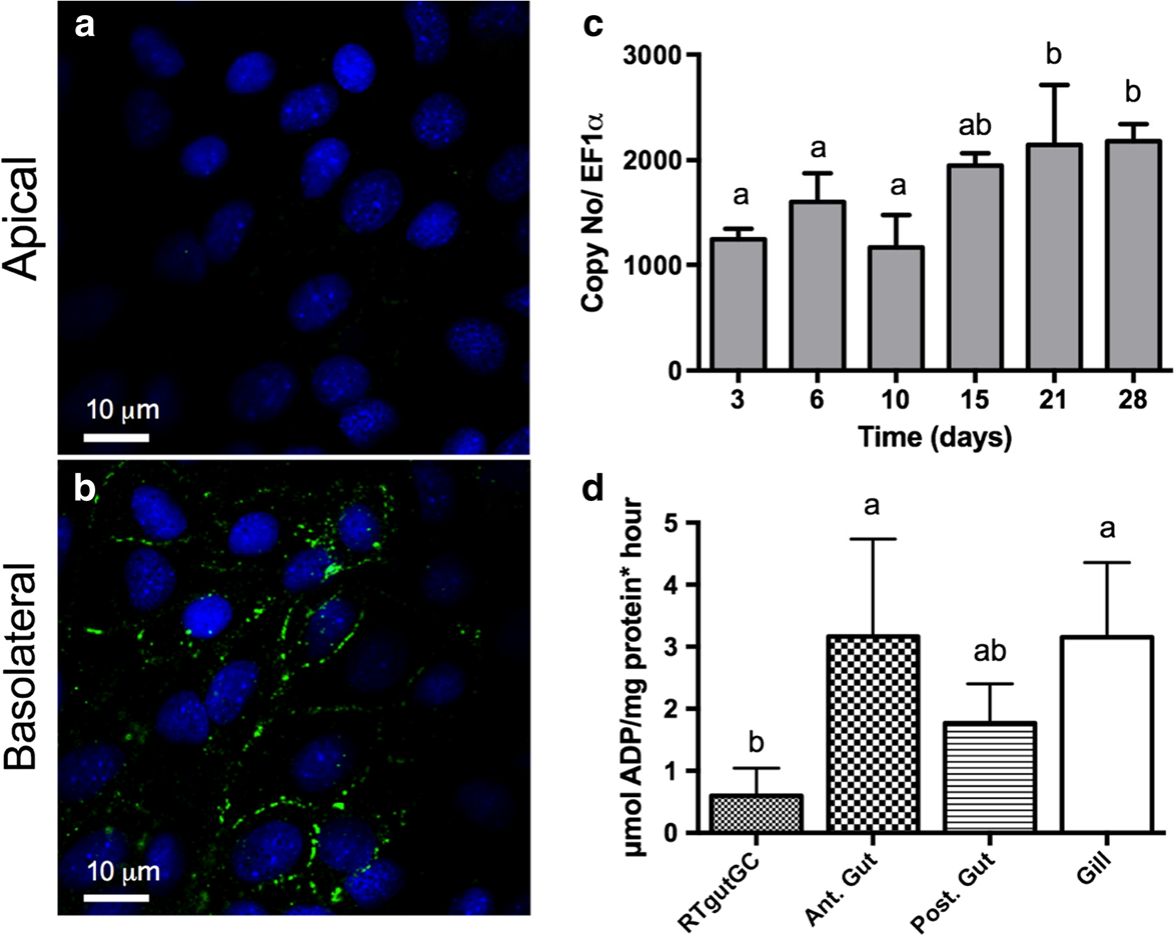
Na^+^/K^−^-ATPase (NKA) localization, mRNA abundance and activity in RTgutGC cells. **a**, **b** Immunocytochemical analysis of RTgutGC cells grown for 25 days on a permeable membrane (0.33 cm^2^—inserts of 24-well plates; starting cell density: 62,500 cm^2^). Following fixation and permeabilization, a double staining was applied: NKA (NKA α_1_-subunit antibody coupled with a Alexa Fluor 488, *green*) and nuclei (DAPI; *blue*). Apical and basolateral images were obtained via Z-stack using a Leica SP5 confocal microscope. **c** NKA mRNA levels in RTgutGC cells grown on permeable membranes over time (1.13 cm^2^—inserts of 12-well plates; starting cell density: 62,500 cm^2^). Expression of target gene mRNA was determined by real-time qPCR using gene specific primers and SyBr green intercalation. Target gene copy numbers were normalized to a reference gene, elongation factor 1 alpha (EF1α). Shown are mean ± standard deviation of three inserts run in parallel. **d** NKA activity in RTgutGC cells grown to confluency in a 75-cm^2^ flask and in tissues obtained from a rainbow trout of a UK fish farm: anterior intestine (*Ant. Gut*), posterior intestine (*Post. Gut*) and gill tissue. *Bars* are means ± standard deviations derived from 11 different preparations for RTgutGC cells, three replicate preparations for Ant. Gut and Post. Gut and four preparations from the gill, (i.e. three to four independent enzyme determinations from the same tissue sample, respectively). *Bars bearing different letters* are significantly different (*p* < 0.05, ANOVA, Tukey test)

Polarization of the cells can also be observed based on the localization of the Na^+^/K^+^-ATPase (NKA), which is expressed basolaterally (Fig. 4a, b), as would be expected in vivo. Moreover, NKA mRNA abundance levels in cells cultured on inserts were consistently detected, reaching stable levels from about day 15 on (Fig. 4c). We were also able to confirm NKA enzyme activity in RTgutGC cells, but this required conventional flask cell culture to obtained sufficient material. As can be seen in Fig. 4d, NKA enzyme activity levels are easily discernible although they are between three to six times lower than in samples of rainbow trout intestine and gill tissue.

**Fig. 5:**
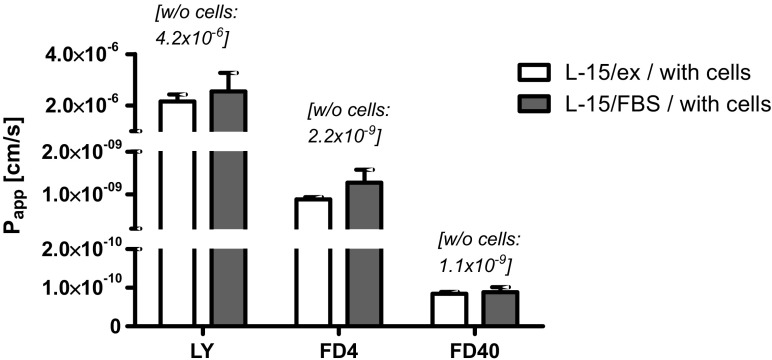
Permeability of the RTgutGC cell culture, grown on permeable support, assessed by determining apparent permeation coefficients for three fluorescent molecules: Lucifer Yellow (LY), Dextran 4000 (FD4) and Dextran 40000 (FD40). Cells were cultured on permeable membranes for 21 to 28 days (1.13 cm^2^—inserts of 12-well plates; starting cell density: 62,500 cm^2^); then, the medium was changed to L-15/FBS or L-15/ex, both under symmetrical conditions, and transfer of the molecules was measured over time. Apparent permeability coefficients were calculated as described by Hubatsch et al. (2007). Each *bar* represents the mean ± standard deviation of at least three independent preparations. *t* test for apparent permeation coefficients in L-15/ex vs. L-15/FBS medium revealed non-significant differences (*p* < 0.05). The *numbers in the brackets above each pair of bars* are the average apparent permeation coefficients calculated for the permeable membrane alone (without cells)

The RTgutGC cell monolayer clearly acts as a barrier for apical to basolateral permeation of fluorescent model molecules, with a decrease in permeation as the size of the fluorescent molecules increases (Fig. 5). The medium itself (L-15/ex vs. L-15/FBS) has no impact on permeability (*t* test, *p* < 0.05).

### Responses of cells to different stimuli

**Fig. 6:**
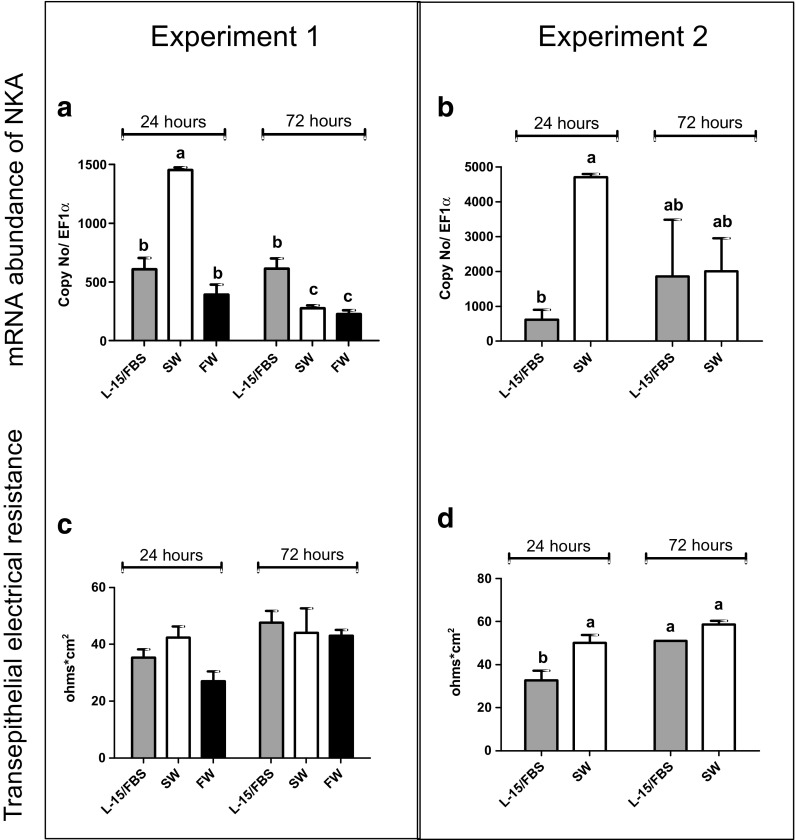
Response of RTgutGC cells to media of different ionic strength mimicking the intestinal environment on saltwater (SW) or freshwater (FW) exposure. Cells were cultured for 21 days (1.13 cm^2^—inserts of 12-well plates; starting cell density: 62,500 cm^2^) before challenging them with the different media apically. The basolateral medium was in all cases L-15/FBS. Two independently performed experiments are shown side by side. **a**, **b** mRNA abundance of NKA 24 and 72 h after changing the medium in the apical chamber to either L-15/FBS (*grey bars*), SW (*white bars*) or FW (*black bars*). Target gene copy numbers were normalized to a reference gene, elongation factor 1 alpha (EF1α). **c**, **d** Blank normalized TEER values measured after 24 and 72 h of exposure. Values are mean ± standard deviation of three to six technical replicates (inserts) for each of the two experiments. *Bars bearing different letters* are significantly different (*p* < 0.05, 2 way ANOVA, Bonferroni test)

To explore the response of the intestinal cell layer to a physiological stimulus, i.e. increased salinity as would be expected during seawater acclimation, 21-day-old cultures were exposed to a freshwater (FW) or a saltwater (SW) buffer on the apical side for up to 72 h. Despite varying levels of NKA mRNA abundance in the two independent cell preparations, which we show side by side, a significant upregulation in response to SW was consistently observed after 24 h (Fig. 6a/b). No impact on mRNA levels occurred upon exposure to FW (Fig. 6a). However, the NKA mRNA induction by SW was transient: comparable or lower levels to L-15/FBS or to FW were found for SW (Fig. 6a/b) after 72 h of exposure. Similarly, TEER values were increased transiently after 24 h, to return to control levels at 72 h. This effect, however, was significant in only one of the two experiments performed (Fig. 6c/d).

**Fig. 7:**
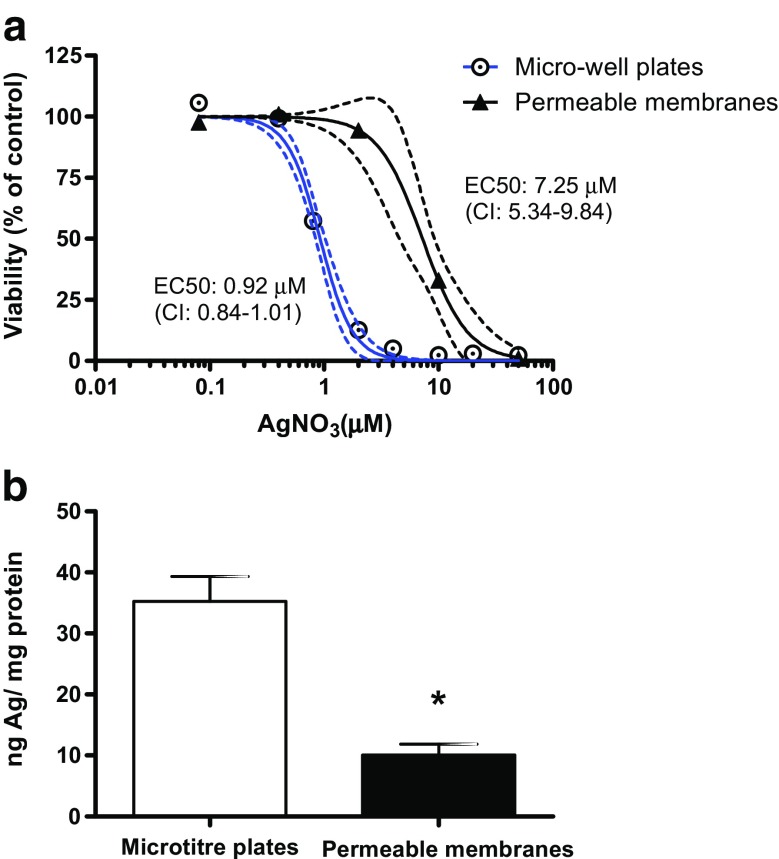
Toxicity and accumulation of silver after 24 h exposure to RTgutGC cells grown as monolayer either on solid support in conventional micro-well plates or on permeable membranes. **a** Cell viability upon exposure to silver as measured by Alamar Blue. Exposure was done on confluent monolayers obtained after 48 h of cell culture in 12-well plates (*circles*) or after 21 days in inserts of 6-well plates (*triangles*) as described in “Material and methods”. Data shown are means and 95% confidence intervals (*dashed lines*) of three independent experiments, each containing three wells/inserts. EC50s (effective concentrations causing a 50% decline of cell viability) were determined by the non-linear regression sigmoidal dose-response curve fitting module using the Hill slope equation. **b** Silver content in cells after exposure to AgNO_3_ for 24 h. Prior to exposure, cells were cultured on solid support in 12-well micro-well plates (*white bar*) or in 6-well plates on permeable membranes (*black bar*). After exposure, cells were dislodged and silver content analysed by ICP-MS. Silver content in the different well set-ups was normalized to total protein content based on the Lowry assay. Shown are means ± standard deviations of three independent experiments, each containing three wells/inserts. The *asterisk* denotes a significant difference in silver accumulation based on *t* test (*p* < 0.05)

To test the response of the cells to a toxicant and evaluate the capacity of RTgutGC cells to process intracellular silver, we exposed the cells for 24 h to silver ions in the form of AgNO_3_ and compared the impact on cell viability to that observed in cells grown and exposed the conventional way in micro-well plates. Based on the effect concentrations causing a 50% reduction of cell viability (EC50 values based on nominally added AgNO_3_), cells grown on inserts were about eightfold less sensitive (Fig. 7a). Quantification of the intracellular silver on exposure to a non-toxic concentration (400 nM AgNO_3_) revealed that the cells in the inserts contain about threefold less silver/milligram protein than cells exposed in micro-well plates (Fig. 7b).

## Discussion

When presenting the first characterization of the RTgutGC cell line, Kawano et al. (2011) proposed that this intestinal cell line may have the potential to become the fish equivalent of the Caco-2 cell line, which is intensively used as human model of pharmacokinetics of drugs and other chemicals (Sun et al. 2008). Our results are in support of this proposal; moreover, in contrast to the Caco-2 cell line, which was derived from a human colon carcinoma, RTgutGC cells were initiated from a healthy rainbow trout.

Establishment of the RTgutGC cell line as a single monolayer on permeable insert membranes leads to formation of a functional epithelium that accommodates transepithelial electrical resistance (TEER) levels of up to 50 Ω × cm^2^ (this study; Geppert et al. 2016). According to the classification presented by Claude and Goodenough (1973), these values are representative of a “leaky” epithelium. Indeed, fish intestinal epithelia have mostly been classified as leaky where resistance is seen as a measure of paracellular permeability (Loretz 1995). For Atlantic salmon (*Salmo salar*) adapted to freshwater, TEER values between 30 and 150 Ω × cm^2^ have been reported (Sundell et al. 2003); thus, the RTgutGC cell line appears to closely reflect the in vivo transepithelial resistance in salmonids. The cell line was initiated from the distal portion of a rainbow trout intestine where TEER is generally found to be higher than in the proximal part—a physiological feature thought to have evolved from lower nutrient but higher bacterial levels toward the distal end (Jutfelt 2011; Sundell and Sundh 2012). Whether such a tightening can be induced in vitro under pre-defined culture conditions will be an interesting avenue for future investigations.

One condition that has been found in vivo to lead to higher TEER values in the proximal and distal fish intestine is saltwater adaptation (Sundell et al. 2003; Sundell and Sundh 2012). Indeed, the RTgutGC cells responded to the high ionic strength SW buffer with a slight increase in TEER values. In vivo, the reported increase in TEER is more marked (Sundell et al. 2003). This observation, however, might be due to the cortisol response that accompanies the increase in TEER due to SW acclimation (Sundell et al. 2003; Taylor et al. 2007).

The TEER is one functional parameter that characterizes epithelia. Another typical feature is the formation of tight junction proteins. To regulate transepithelial transport, tight junctions are located apically in the mature epithelium. Indeed, apical localization was confirmed in RTgutGC cells by electron microscopy as well as by antibody-based staining of the tight junction protein ZO-1. Apical localization of ZO-1 by immunocytochemistry has previously been demonstrated in primary rainbow trout gill epithelia likewise grown on commercially available permeable membranes (Schnell et al. 2016; Walker et al. 2007). Movement of the ZO-1 protein was seen in the RTgutGC epithelium over time: while it was not detectable apically on day one, apical signals increased, reaching a maximum within 21 days. In further support of the maturation of a polarized epithelium in vitro, we observed the development of a profound actin network in the cells. Initially, actin was only detectable basolaterally but with time, co-localized in a circumferential ring with ZO-1, in addition to forming so-called stress fibres basolaterally, attaching the cells to the surface of the permeable support. Very similar features but focussing on another tight junction protein, Claudin 28b, were described for primary rainbow trout gill cell cultures seeded as monolayers in culture inserts (Sandbichler et al. 2011). It is also interesting to note that the mature gill cell cultures were described as being 10–15 μM in height (Sandbichler et al. 2011). This is similar to the height of the RTgutGC epithelium after cell proliferation and maturation for about 21 days.

Another important characteristic of polarized intestinal epithelial cells is the expression of NKA, which plays an important role for several transepithelial transport processes concerned with nutrient uptake and ion regulation (Grosell et al. 2007; Marshall and Grosell 2006). NKA is present and active in the RTgutGC cell line. As the epithelium matured, mRNA expression levels increased over a period of about 2 weeks before remaining stable. We measured the NKA enzyme activity for the first time in a rainbow trout cell line even though it was not yet possible to do this in RTgutGC cells taken from insert cultures because of the high amount of material needed. Comparison with levels measured in parallel in rainbow trout intestinal and gill tissues, obtained from a completely different source of rainbow trout, revealed four to seven times lower activity in the RTgutGC cells grown in conventional cell culture flasks. However, other reports have presented NKA activity values in tissues very close to that measured here in RTgutGC cells, likely reflecting interindividual variability as well as the physiological status of the animals from which the tissue was taken. Overall, reported NKA levels in salmonids in gill or intestine range between ~0.5 μmol ADP mg protein^−1^ h^−1^ (Sundell et al. 2003; Atlantic salmon, posterior intestine, freshwater adopted) and 40 μmol ADP mg protein^−1^ h^−1^ (Taylor et al. 2007; rainbow trout gill adapting to seawater). The higher levels are measured upon seawater adaptation where augmented NKA activity aids the fish in salt secretion. In support of this process, we found a transient increase in NKA mRNA abundance upon exposure to the SW buffer for 24 h while in RTgutGC exposed for 72 h, mRNA returned to, or even below, control levels. A similar transient increase in NKA mRNA levels has been reported previously as an adaptive response in the intestine of rainbow trout abruptly transferred to 65% saltwater (Grosell et al. 2007). Our proof-of-concept experiment is encouraging for the use of the RTgutGC cells as model to study factors influencing NKA expression specifically and freshwater-seawater adaptations more generally. Importantly, for the RTgutGC epithelial cell system, NKA was localized basolaterally as expected from the in vivo situation (Sundell and Sundh 2012).

Three differently sized and fluorescently marked molecules were studied for their permeation across the RTgutGC epithelial barrier cultured on the insert supports: LY—522 Da; FD4—4000 Da and FD40—40,000 Da. The cells clearly presented a barrier for these molecules, and permeation decreased as molecule size grew. Studies on the permeation of molecules across the fish intestinal barrier in vivo have largely focussed on small molecules with sizes even smaller than LY (e.g. urea—62 Da; erythritol—124 Da; mannitol—184 Da), with the greatest difference in permeation seen for urea (Sundell and Sundh 2012). The apparent permeability coefficients for LY in the RTgutGC model were indeed in the same range as those reported for erythritol and mannitol in freshwater adapted rainbow trout (Sundell and Sundh 2012). Permeability coefficients for FD4 and FD40 where about three orders of magnitude lower for compared to LY. The small difference observed in permeation of FD4 and FD40 indicates that there is a size cut-off for permeation. Opposite to permeation of nanoparticles (Geppert et al. 2016), the pore size of the culture membrane is unlikely to restrict permeation: the 40,000 Da dextran molecule is estimated to be about 9 nm in diameter (see Sigma product description) whereas the pores of the PET permeable membrane are 400 nm in diameter. The dextran size is about 20-fold larger than the frequently reported junctional pore size of 0.4 nm in human-derived cells (Watson et al. 2005), which would suggest transcellular permeation of FD4/40 molecules across the RTgutGC barrier. The specific junctional pore size in the RTgutGC model or in the fish intestine in vivo is not yet known; as well, pore size alone did not explain differences in transepithelial resistance in the human-derived Caco2/T84 cells (Watson et al. 2001). The RTgutGC cell barrier model therefore offers new opportunities to study the regulation of junctional proteins and associated permeation processes under physiological or toxicological conditions with a focus on fish.

Indeed, one of the areas of exploitation of the RTgutGC intestinal model is in environmental toxicology. Our results demonstrate that cells cultured on the permeable membrane are more tolerant to silver ion exposure than cells cultured on regular, solid culture support. One could argue that the greater resistance, i.e. lower sensitivity to silver ion exposure, might be due to a greater cell density on the inserts, where cells were cultured over 21 days compared to 48 h in conventional culture wells. Thus, less silver per cell might per se be available. However, to account for this possibility, we normalized the levels of silver measured in the cells to total protein in the different culture set-ups. On this basis, cells on inserts showed significantly lower intracellular silver accumulation. We therefore propose that these cells are better able to eliminate silver. Silver is known to hijack copper transporters (Behra et al. 2013). The main copper excretory protein, the Cu-ATPase ATP7A, is expressed and functions basolaterally in polarized epithelial cells in mammals (Lutsenko et al. 2008). In this way, the polarized RTgutGC cells may be more effective in removing silver into the basolateral space. Although we could not localize ATP7A in RTgutGC due to the lack of a fish specific antibody, the ATP7A gene is present in fish as well as other vertebrates (Minghetti et al. 2010). Basolateral localization of the NKA indicates the possibility that other ATPases would localize basolaterally allowing a more efficient metal homeostasis.

In conclusion, we here present development and initial characterization of the first in vitro intestinal barrier model for fish. Built from a cell line derived from the intestine of a rainbow trout (Kawano et al. 2011), this model offers many new opportunities for mechanistic investigations into the roles and functions of the intestine in fish. We demonstrate several key features that match the characteristics of the fish intestinal epithelium in vivo, such as the extent of transepithelial electrical resistance and the typical localization of proteins featuring polarization of the epithelial cells, clearly observed within 21 culture days. A focus for further model development could be to attempt the stimulation of microvilli formation and the characterization of mucus production. However, as it stands already now, this model holds promise to address a variety of processes that is difficult to disentangle in vivo, such as cellular mechanisms of physiological and pathological responses, immune function, nutrient uptake and ability of intestinal cells to act as a barrier for toxicants.

## Abbreviations

FW: Freshwater
L-15: Leibovitz’s culture medium
NKA: Na^+^/K^+^-ATPase
RTgutGC: Intestinal cell line from rainbow trout (rainbow trout gut Germany-Canada)
SW: Seawater
TEER: Transepithelial electrical resistance
ZO-1: Zona occludens 1 (a tight junction protein)

## References

[CR1] Behra R, Sigg L, Clift MJD, Herzog F, Minghetti M, Johnston B, Petri-Fink A, Rothen RB (2013). Bioavailability of silver nanoparticles and ions: from a chemical and biochemical perspective. J R Soc Interface.

[CR2] Burke J, Handy RD (2005). Sodium-sensitive and -insensitive copper accumulation by isolated intestinal cells of rainbow trout *Oncorhynchus mykiss*. J Exp Biol.

[CR3] Bury NR, Schnell S, Hogstrand C (2014). Gill cell culture systems as models for aquatic environmental monitoring. J Exp Biol.

[CR4] Claude P, Goodenough DA (1973). Fracture faces of zonulae occludentes from “tight” and “leaky” epithelia. The Journal of Cell Biol.

[CR5] Clevers H (2013). The intestinal crypt, a prototype stem cell compartment. Cell.

[CR6] Ganassin RC, Schirmer K, Bols NC, Bullock G, Bunton TE, Ostrander G (2000). Experimental models: cell/tissue cultures—methods for the use of fish cell and tissue culture as model systems in basic and toxicology research. The handbook of experimental animals, laboratory fish.

[CR7] Genz J, Esbaugh AJ, Grosell M (2011). Intestinal transport following transfer to increased salinity in an anadromous fish (*Oncorhynchus mykiss*). Comp Biochem Physiol A Mol Integr Physiol.

[CR8] Geppert M, Sigg L, Schirmer K (2016). A novel two-compartment barrier model for investigating nanoparticle transport in fish intestinal epithelial cells. Environmental Science: Nano.

[CR9] Grosell M, Gilmour KM, Perry SF (2007). Intestinal carbonic anhydrase, bicarbonate, and proton carriers play a role in the acclimation of rainbow trout to seawater. Am. J. Physiol. Regul. Integr. Comp Physiol.

[CR10] Grosell M, Grosell M, Farrell AP, Brauner CJ (2011). The multifunctional gut of fish.

[CR11] Handy RD, Musonda MM, Phillips C, Falla SJ (2000). Mechanisms of gastrointestinal copper absorption in the African walking catfish: copper dose-effects and a novel anion-dependent pathway in the intestine. J Exp Biol.

[CR12] Hogstrand C, Wood CM, Bury NR, Wilson RW, Rankin JC, Grosell M (2002). Binding and movement of silver in the intestinal epithelium of a marine teleost fish, the European flounder (*Platichthys flesus*). Comp. Biochem. Physiol.—C Toxicol Pharmacol.

[CR13] Hubatsch I, Ragnarsson EGE, Artursson P (2007). Determination of drug permeability and prediction of drug absorption in Caco-2 monolayers. Nat Protoc.

[CR14] Jutfelt F (2011). Barrier function of the gut. Encyclopedia of Fish Physiology 2011.

[CR15] Kawano A, Haiduk C, Schirmer K, Hanner R, Lee LEJ, Dixon B, Bols NC (2011). Development of a rainbow trout intestinal epithelial cell line and its response to lipopolysaccharide. Aquat Nutr.

[CR16] Kültz D (2015). Physiological mechanisms used by fish to cope with salinity stress. J Exp Biol.

[CR17] Kwong RWM, Niyogi S (2009). The interactions of iron with other divalent metals in the intestinal tract of a freshwater teleost, rainbow trout (*Oncorhynchus mykiss*). Comp Biochem Physiol—C Toxicol Pharmacol.

[CR18] Kwong RW, Niyogi S (2012). Cadmium transport in isolated enterocytes of freshwater rainbow trout: interactions with zinc and iron, effects of complexation with cysteine, and an ATPase-coupled efflux. Comp Biochem Physiol C Toxicol Pharmacol.

[CR19] Loretz CA, Chris MW, Trevor JS (1995). Electrophysiology of ion transport in teleost intestinal cells. Fish physiology.

[CR20] Lutsenko S, Gupta A, Burkhead JL, Zuzel V (2008). Cellular multitasking: the dual role of human Cu-ATPases in cofactor delivery and intracellular copper balance. Arch Biochem Biophys.

[CR21] Marshall WS, Grosell M, Evans DH, Claiborne JB (2006). Ion transport, osmoregulation, and acid–base balance. The physiology of fishes.

[CR22] McCormick SD (1993). Methods for nonlethal gill biopsy and measurement of Na+, K+-ATPase activity. Can J Fish Aquat Sci.

[CR23] Minghetti M, Schirmer K (2016). Effect of media composition on bioavailability and toxicity of silver and silver nanoparticles in fish intestinal cells (RTgutGC). Nanotoxicology.

[CR24] Minghetti M, Leaver MJ, George SG (2010). Multiple Cu ATPase genes are differentially expressed and transcriptionally regulated by Cu exposure in sea bream, *Sparus aurata*. Aquat Toxicol.

[CR25] O’Brien J, Wilson I, Orton T, Pognan F (2000). Investigation of the Alamar Blue (resazurin) fluorescent dye for the assessment of mammalian cell cytotoxicity. Eur J Biochem.

[CR26] Sambuy Y, De Angelis I, Ranaldi G, Scarino ML, Stammati A, Zucco F (2005). The Caco-2 cell line as a model of the intestinal barrier: influence of cell and culture-related factors on Caco-2 cell functional characteristics. Cell Biol Toxicol.

[CR27] Sandbichler AM, Egg M, Schwerte T, Pelster B (2011). Claudin 28b and F-actin are involved in rainbow trout gill pavement cell tight junction remodeling under osmotic stress. J Exp Biol.

[CR28] Schirmer K, Chan AGJ, Greenberg BM, Dixon DG, Bols NC (1997). Methodology for demonstrating and measuring the photocytotoxicity of fluoranthene to fish cells in culture. Toxicol in Vitro.

[CR29] Schnell S, Stott LC, Hogstrand C, Wood CM, Kelly SP, Pärt P, Owen SF, Bury NR (2016). Procedures for the reconstruction, primary culture and experimental use of rainbow trout gill epithelia. Nat Protoc.

[CR30] Sun H, Chow ECY, Liu S, Du Y, PangSun KS (2008). The Caco-2 cell monolayer: usefulness and limitations. Expert Opin Drug Metab Toxicol.

[CR31] Sundell KS, Sundh H (2012). Intestinal fluid absorption in anadromous salmonids: importance of tight junctions and aquaporins. Front Physiol.

[CR32] Sundell K, Jutfelt F, Olsen R, Sandblom E, Hansen T, Bjornsson BT (2003). Intestinal transport mechanisms and plasma cortisol levels during normal and out-of-season parr–smolt transformation of Atlantic salmon, *Salmo salar*. Aquaculture.

[CR33] Sundh H, Nilsen TO, Lindström J, Hasselberg-Frank L, Stefansson SO, McCormick SD, Sundell K (2014). Development of intestinal ion-transporting mechanisms during smoltification and seawater acclimation in Atlantic salmon *Salmo salar*. J Fish Biol.

[CR34] Taylor JF, Needham MP, North BP, Morgan A, Thompson K, Migaud H (2007). The influence of ploidy on saltwater adaptation, acute stress response and immune function following seawater transfer in non-smolting rainbow trout. Gen Comp Endocrinol.

[CR35] Walker PA, Bury NR, Hogstrand C (2007). Influence of culture conditions on metal-induced responses in a cultured rainbow trout gill epithelium. Environ Sci Technol.

[CR36] Watson CJ, Rowland M, Warhurst G (2001). Functional modeling of tight junctions in intestinal cell monolayers using polyethylene glycol oligomers. Am J Physiol Cell Physiol.

[CR37] Watson CJ, Hoare CJ, Garrod DR, Carlson GL, Warhurst G (2005). Interferon-gamma selectively increases epithelial permeability to large molecules by activating different populations of paracellular pores. J Cell Sci.

